# Supplementary material for: Are There Differences in Postural Control and Muscular Activity in Individuals with COPD and with and Without Sarcopenia?
Source: Adv Respir Med. 2025 Feb 18;93(1):5. doi: 10.3390/arm93010005 (PMC11851567; doi:10.3390/arm93010005)
Supplement: Supplementary file 1 [file arm-93-00005-s001.zip › arm-3224314-supplementary.pdf]

Table S 1: Correlation matrix between force platform variables and the percentage of muscle activation during various tasks in individuals with sarcopenia									
Variables	TA	GA	VM	GM	RA	ES	INT	SCM	SC
**Bipedal eyes opened**									
COP-a	0,33333333	0,03030303	-0,5030303	-0,47416632	0,61212121	0,32121212	0,15151515	-0,34545455	-0,76596099
Vel-AP	0,33333333	-0,00606061	-0,55151515	-0,48632443	0,61212121	0,21212121	0,24848485	-0,53939394	-0,76596099
Vel-ML	0,35258522	-0,00607906	-0,49848255	-0,47560976	0,62006365	0,32826899	0,10334394	-0,3404271	-0,76829268
Amp-AP	-0,04863244	0,40121766	0,37082238	0,38414634	-0,49848255	0,09726489	-0,06686961	0,27963655	-0,04878049
Amp-ML	0,109423	0,37082238	0,12766016	0,43292683	-0,30395277	0,23100411	0,07902772	0,18237166	-0,25
**Bipedal eyes closed**									
COP-a	-0,18237166	-0,08510678	0,41945483	-0,18845072	0,43769199	0,35365854	-0,06079055	-0,1641345	-0,47416632
Vel-AP	-0,03647433	-0,01215811	0,27355749	0,02431622	0,34650616	0,32317073	-0,11550205	-0,07294867	-0,58966838
Vel-ML	-0,11515152	-0,00606061	0,45454545	0,12727273	0,46666667	0,35258522	-0,2	0,07878788	-0,43030303
Amp-AP	-0,13981828	0,44377105	-0,20668788	0,31003183	0,20060883	0,001	0,24924127	0,30395277	-0,26747844
Amp-ML	-0,12727273	0,63636364	-0,16363636	0,23636364	0,46666667	-0,1641345	0,46666667	0,32121212	-0,00606061
**One-legged stance**									
COP-a	0,48571429	-0,08571429	0,71428571	-0,2	0,54285714	-0,02857143	0,6	0,02857143	0,31428571
Vel-AP	0,31428571	-0,25714286	0,54285714	0,08571429	0,77142857	0,31428571	0,77142857	0,08571429	0,2
Vel-ML	0,82857143	-0,02857143	0,71428571	-0,25714286	0,94285714	-0,2	0,77142857	-0,08571429	0,77142857
Amp-AP	0,48571429	-0,37142857	0,65714286	-0,02857143	0,88571429	0,37142857	0,65714286	0,14285714	0,25714286
Amp-ML	0,54285714	-0,25714286	0,48571429	0,08571429	0,94285714	0,2	0,77142857	0,08571429	0,48571429
**Bipedal on unstable surface**									

COP-a	-0,12727273	-0,46808727	-0,47878788	-0,34545455	-0,15151515	-0,46666667	-0,26747844	-0,26060606	-0,34650616
Vel-AP	-0,13939394	-0,40121766	-0,41818182	-0,23636364	0,13939394	-0,26060606	-0,12158111	-0,22424242	-0,32218994
Vel-ML	-0,30909091	-0,29179466	-0,33333333	-0,41818182	-0,09090909	-0,41818182	0,06079055	-0,40606061	-0,52887782
Amp-AP	-0,22424242	0,06686961	-0,2969697	-0,45454545	0,33333333	0,07878788	0,06686961	-0,13939394	0,12158111
Amp-ML	0,21276694	-0,39634146	-0,57143121	-0,71124949	0,14589733	0,00607906	-0,20121951	-0,45592916	-0,4054878

COP-area: center of pressure displacement area; Vel-AP: center of pressure displacement velocity in anterior-posterior direction; Vel-ML: center of pressure displacement velocity in medial-lateral direction; Amp-AP amplitude of the movement of COP in antero-posterior direction; Amp-ML amplitude of the movement of COP in medial-lateral direction; TA: tibialis anterior; GA: gastrocnemius, VM: vastus medialis; GM: gluteus medius; ES: erector spinae; RA: rectus abdominis; INT: external intercostal; SCM: sternocleidomastoid; SC: scalene.

Table S2: Correlation matrix between force platform variables and the percentage of muscle activation during various tasks in individuals without sarcopenia

Variables	TA	GA	VM	GM	RA	ES	INT	SCM	SC
**Bipedal eyes opened**									
COP-a	-0,03510584	0,17510331	-0,00376223	-0,45372473	-0,23024837	-0,02258186	0,17908207	-0,24087318	-0,07298723
Vel-AP	-0,28187923	0,29700413	-0,07596841	-0,17976684	-0,21511848	0,13920241	-0,07747274	-0,11738149	0,11056789
Vel-ML	0,5624355	0,4481157	0,16021062	-0,21436632	-0,05114705	-0,23325809	0,31214745	-0,34198646	-0,23016173
Amp-AP	0,39132685	0,33677686	0,25874391	0,09176383	0,26024823	-0,32656132	0,04663408	-0,14484575	-0,1444152
Amp-ML	0,42621259	0,54620555	0,35338346	-0,0075188	0,11578947	-0,17976684	0,22556391	-0,07897706	-0,2481203
**Bipedal eyes closed**									
COP-a	-0,08569955	0,29442149	0,17751035	-0,04176072	-0,13162844	-0,23843552	0,14145974	0,18660647	0,14892818
Vel-AP	-0,15178113	0,11518595	0,14215872	-0,06734387	-0,23693119	-0,33396016	-0,23212942	-0,10308503	0,27002634
Vel-ML	-0,0753743	0,12654959	0,21887929	-0,12979684	-0,13689358	0,0842422	0,08502634	0,086155	0,03384731

Amp-AP	0,20960251	-0,31301653	0,4054156	-0,02859293	0,04287326	-0,49793159	-0,58765989	-0,09217457	-0,03309515
Amp-ML	0,0753354	-0,25606612	0,33082707	-0,19932307	-0,00150376	-0,54586466	-0,58894325	-0,00376081	0,01804511
**One-legged stance**									
COP-a	-0,52747253	-0,14835165	-0,12857143	0,20357143	-0,18214286	0,39285714	0,11071429	0,15357143	0,23214286
Vel-AP	-0,00549451	0,05494505	-0,40357143	-0,21428571	-0,18214286	-0,03571429	-0,45	-0,21071429	-0,25714286
Vel-ML	0,31086687	0,42090824	-0,03395891	0,15728335	-0,45040232	0,03395891	-0,15013411	-0,21983923	-0,11438789
Amp-AP	-0,53846154	-0,20879121	-0,21785714	0,375	-0,08928571	0,06428571	0,02857143	0,11071429	0,35
Amp-ML	-0,47802198	-0,12087912	-0,29642857	0,25	-0,1	0,16428571	-0,04285714	0,01428571	0,18571429
**Bipedal on unstable surface**									
COP-a	-0,30443756	-0,09184727	-0,07067669	-0,33383459	0,24905951	0,27218045	0,69575033	0,31666042	-0,18345865
Vel-AP	-0,29411765	0,02786378	-0,22940957	-0,32192556	-0,05494919	-0,05039489	0,1489842	-0,11437171	0,07897706
Vel-ML	0,23140508	-0,06095045	0,44319049	-0,29194892	-0,12349398	0,33107609	0,24576592	-0,01279639	-0,2422875
Amp-AP	-0,16012405	0,16322323	-0,20015055	-0,46576386	0,32718373	-0,04665163	-0,32216788	-0,03048551	-0,13468777
Amp-ML	0,08673207	0,00309757	0,00225649	-0,53553971	0,48137	-0,07972923	-0,24529722	0,11963883	-0,3271907
COP-area: center of pressure displacement area; Vel-AP: center of pressure displacement velocity in anterior-posterior direction; Vel-ML: center of pressure displacement velocity in medial-lateral direction; Amp-AP amplitude of the movement of COP in antero-posterior direction; Amp-ML amplitude of the movement of COP in medial-lateral direction; TA: tibialis anterior; GA: gastrocnemius, VM: vastus medialis; GM: gluteus medius; ES: erector spinae; RA: rectus abdominis; INT: external intercostal; SCM: sternocleidomastoid; SC: scalene.									

Table S3: Correlation between static balance in force platform with body composition and functional variables

**Bipedal eyes opened**	COP-a		Vel-AP		Vel-ML		Amp-AP		Amp -ML	
	r	p	r	p	r	p	r	p	r	p
BMI	-0.159	0.354	-0,630	0.000*	-0,640	0.000*	0.013	0.939	-0.139	0.418
FFMI	-0.190	0.283	-0,611	0.000*	-0,605	0.000*	-0.088	0.622	-0.227	0.196

SMI	-0.170	0.359	-0,709	0.000*	-0,575	0.001*	0.073	0.696	-0.046	0.804
FMI	-0.046	0.797	-0.290	0.096	-0.315	0.070	0.080	0.652	0.030	0.867
Brief-BEST	-0.135	0.427	-0,376	0.022*	-0,442	0.006*	-0.071	0.675	-0.249	0.138
TUG	0.291	0.080	0.291	0.081	0.315	0.058	0,337	0.041*	0.200	0.236
HGS	-0.346	0.053	-0,443	0.011*	-0,450	0.010*	-0.129	0.481	-0.251	0.166
4MGS	0.027	0.896	-0,483	0.012*	-0,417	0.034*	-0.106	0.608	-0.007	0.971
**Bipedal eyes closed**	COP-a		Vel-AP		Vel-ML		Amp-AP		Amp -ML	
	r	p	r	p	r	p	r	p	r	p
BMI	-0.087	0.613	-0.036	0.833	-0,475	0.003*	-0.002	0.993	-0.128	0.458
FFMI	-0.154	0.384	-0.034	0.848	-0,460	0.006*	-0.152	0.391	-0.219	0.213
SMI	-0.169	0.363	-0.162	0.385	-0,462	0.009*	-0.075	0.688	-0.115	0.538
FMI	0.033	0.853	-0.026	0.885	-0.227	0.197	0.128	0.470	0.057	0.749
Brief-BEST	-0.047	0.783	0.213	0.205	-0.140	0.408	-0.061	0.720	-0.118	0.486
TUG	0.242	0.149	0.073	0.667	0.269	0.107	0,348*	0.035	0.180	0.286
HGS	-0.289	0.109	-0.180	0.325	-0,446	0.011*	-0.203	0.266	-0.245	0.176
4MGS	0.067	0.744	0.295	0.143	-0.021	0.920	-0.019	0.927	0.025	0.903
**One-legged stance†**	COP-a		Vel-AP		Vel-ML		Amp-AP		Amp -ML	
	r	p	r	p	r	p	r	p	r	p
BMI	-0.136	0.490	-0.119	0.547	0.006	0.976	0.189	0.335	-0.086	0.665
FFMI	-0.110	0.592	0.004	0.985	0.060	0.772	0.185	0.365	-0.140	0.496
SMI	-0.057	0.791	0.135	0.528	0.197	0.357	0.297	0.158	-0.035	0.869
FMI	-0.174	0.395	-0.202	0.322	-0.059	0.775	0.123	0.549	-0.042	0.838
Brief-BEST	-0.351	0.067	-0.256	0.189	-0.253	0.194	0.052	0.792	0.021	0.916
TUG	0.274	0.158	0.033	0.866	-0.007	0.974	-0.023	0.908	-0.054	0.785
HGS	-0.168	0.432	0.086	0.689	0.221	0.300	-0.030	0.889	0.158	0.461
4MGS	-0.287	0.219	-0.385	0.094	-0.321	0.168	0.145	0.541	0.148	0.533
**Bipedal on unstable surface**	COP-a		Vel-AP		Vel-ML		Amp-AP		Amp -ML	
	r	p	r	p	r	p	r	p	r	p
BMI	-0.257	0.130	0.050	0.770	0.051	0.768	-0,422	0.010*	-0,419	0.011*
FFMI	-0.316	0.068	0.043	0.808	0.044	0.805	-0,588	0.000*	-0,593	0.000*
SMI	-0.220	0.234	0.041	0.826	0.053	0.778	-0.262	0.155	-0.293	0.110
FMI	-0.057	0.747	0.020	0.909	0.020	0.908	-0.010	0.956	0.010	0.956
Brief-BEST	-0.212	0.207	0.226	0.179	0.207	0.218	-0,331*	0.045	-0.300	0.071

TUG	0.239	0.154	0.093	0.583	0.113	0.505	0.186	0.272	0.148	0.383
HGS	-0,377	0.033*	-0.140	0.445	-0.140	0.444	-0,414	0.018*	-0,393	0.026*
4MGS	0.023	0.911	0.355	0.075	0.360	0.071	-0.185	0.366	-0.042	0.838

Brief-BEST: Brief Balance Evaluation Systems Test; BMI: body mass index; COPD: chronic obstructive pulmonary disease; COP-area: center of pressure displacement area; FFMI: fat-free mass index; HGS: handgrip strength; TUG: Timed Up and Go test; SMI: skeletal muscle mass index; 4MGS: 4-meter gait speed; Vel-AP: center of pressure displacement velocity in anterior-posterior direction; Vel-ML: center of pressure displacement velocity in medial-lateral direction; Amp-AP amplitude of the movement of COP in antero-posterior direction; Amp-ML amplitude of the movement of COP in medial-lateral direction. † Analyse done with n=20. * statistical significate $P < 0.05$

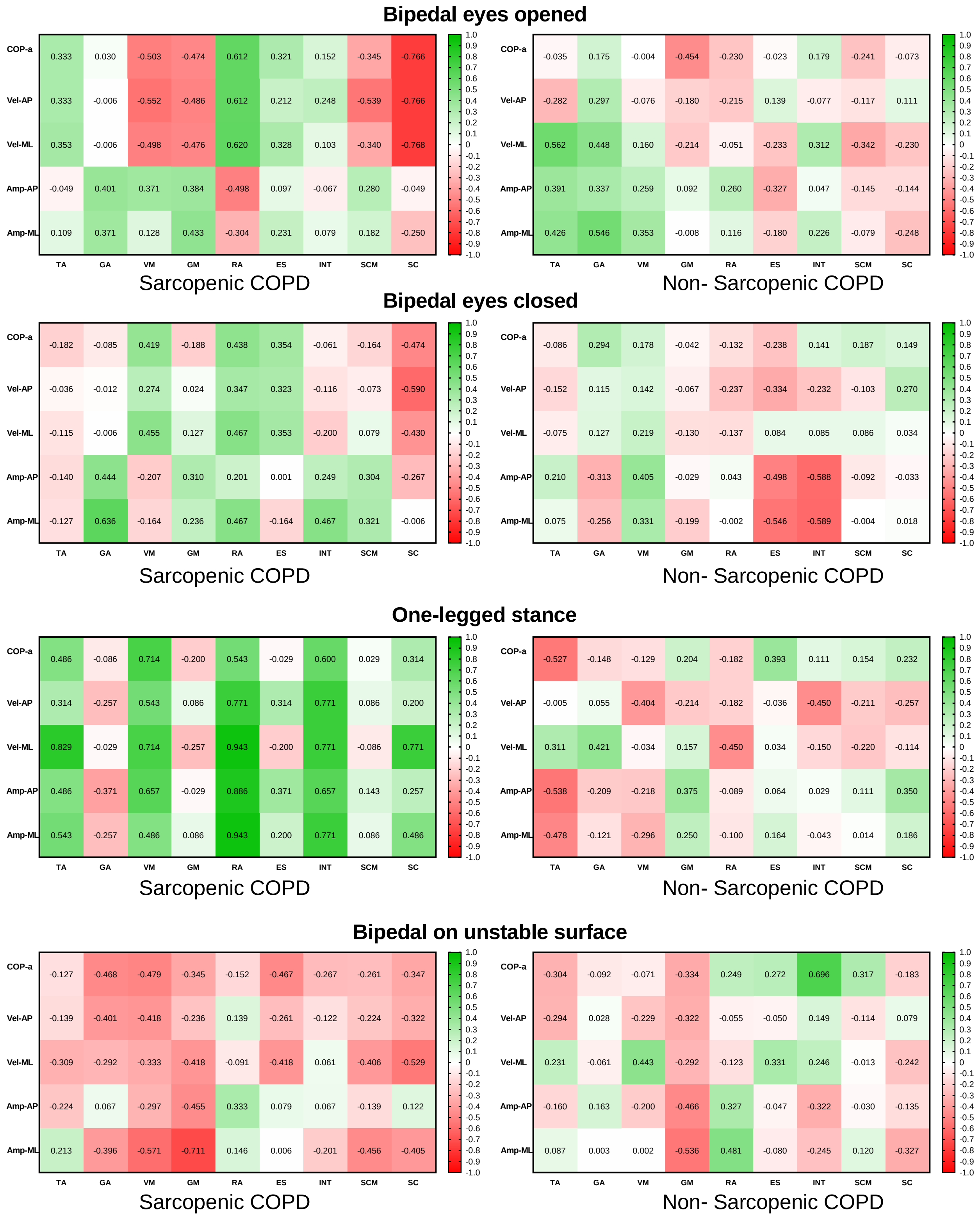

Figure S1. Correlations between force platform variables and the percentage of muscle activation during various tasks in individuals with and without sarcopenia. More intense colors (red or green) for more extreme correlations (close to 1 or -1 respectively), and correlations not significantly different from 0 are represented by a white box. In addition, positive correlations (green) indicate that an in-crease in instability is also associated with greater muscle activation. Negative correlations (red) suggest that greater instability is related to lower muscle activation. TA: tibialis anterior; GA: gastrocnemius, VM: vastus medialis; GM: gluteus medius; ES: erector spinae; RA: rectus abdominis; INT: external intercostal; SCM: sternocleidomastoid; SC: scalene; COP-area: center of pressure displacement area; Vel-AP: center of pressure displacement velocity in anterior-posterior direction; Vel-ML: center of pressure displacement velocity in medial-lateral di-rection; Amp-AP amplitude of the movement of COP in antero-posterior direction; Amp-ML amplitude of the movement of COP in medial-lateral direction.
